# Healthcare poverty-inequality and government quick fixes

**DOI:** 10.1192/bjb.2020.139

**Published:** 2021-02

**Authors:** Claire Hilton

**Affiliations:** Historian in Residence, Royal College of Psychiatrists. email: claire.hilton6@gmail.com

I welcome the editorial by Peter Byrne and Adrian James^1^ on poverty-inequality, and their note about lessons from history. Reports indicating the damage to health caused by poverty-inequality in Britain go back at least as far as Benjamin Seebohm Rowntree's study of York around 1900, Julian Tudor Hart's ‘inverse care law’ in 1971, and the government-commissioned and suppressed Black Report of 1980.^2^ The narrative of governments abandoning some of the most deprived and vulnerable people in society is ongoing.

As Byrne and James point out, people with severe mental illness today have an additional layer of disadvantage, a ‘lower status conferred on them’, a state of ‘subcitizenship’, due to stigma and marginalisation, associated with societal and government disinclination to resource care for them. This too is long term. In 1908, psychiatrist William Stoddart^3^ accused the asylum leadership (which had statutory responsibility for the care of mentally unwell people) of having ‘excessively economical tendencies’, neglecting their patients, the subcitizens of their time. This neglect was associated with adverse outcomes, such as excess morbidity and mortality from physical illness: in asylums, the death rate from tuberculosis, a poverty-related potentially preventable disease, was ten times higher than in the community. Then, as now, it was convenient for the authorities to attribute high rates of physical illness to a person's underlying mental disorder, rather than providing resources to allow services to support those patients adequately, whether in the asylums of the past or in the community today.

Government bodies have repeatedly sought the cheapest short-term measures for managing mental disorders, overlooking social and environmental root causes of the problems and failing to consider longer-term health and social benefits of adequate resourcing. Sometimes these principles extend to public health more generally. Perhaps the most outstanding recent demonstration of a quick-and-cheap government fix was the advice at the beginning of the COVID-19 pandemic for everyone to take vitamin D, based on the finding of high mortality from COVID-19 in Black and minority ethnic groups, who are particularly likely to have low levels. If vitamin D has any effect, it appears to be non-specific.^4^ In other words, the quick-and-cheap fix did not work. Rather, COVID-19 deaths, as Byrne and James remind us, are associated with social deprivation, which may also be associated with low vitamin D. Vitamin D won't fix the real problems.
